# Entropy-Based Analysis of Olfactory EEG as a Candidate Biomarker for Early Mild Cognitive Impairment Detection: A Proof-of-Concept Study

**DOI:** 10.3390/bios16090504

**Published:** 2026-09-08

**Authors:** Sabatina Criscuolo, Andrea De Maria, Annarita Tedesco, Pasquale Arpaia, Egidio De Benedetto

**Affiliations:** 1 Institute of Intelligent Industrial Technologies and Systems for Advanced Manufacturing (STIIMA), National Research Council of Italy, 23900 Lecco, Italy; sabatinacriscuolo@cnr.it; 2 Department of Information Technology and Electrical Engineering (DIETI), University of Naples Federico II, 80125 Naples, Italy; andrea.demaria@unina.it (A.D.M.); pasquale.arpaia@unina.it (P.A.); 3 Department of Public Health, University of Naples Federico II, 80145 Naples, Italy; annarita.tedesco@unina.it

**Keywords:** olfactory EEG, mild cognitive impairment, multiscale multivariate multi-frequency entropy, EEG complexity, biomarkers, measurements

## Abstract

A decline in olfactory ability represents one of the earliest signs of Alzheimer’s disease (AD) and can be valuable information for early diagnosis at the stage of mild cognitive impairment (MCI). Nevertheless, the underlying neurophysiological mechanisms of olfactory impairment have not been systematically studied and, so far, have not been applied to make objective diagnoses using electroencephalography (EEG). To fill this gap, this proof-of-concept study investigates the possibility of using olfactory-evoked EEG complexity to discriminate between healthy subjects (HSs) and MCI patients. To this purpose, a publicly available olfactory oddball EEG-recording dataset was considered. First, a strategy for cleaning the EEG signals was implemented and applied, including exclusion of participants, channels, and epochs affected by substantial artifacts and noise. Then, a dedicated preprocessing pipeline was implemented: in particular, the cleaned signals were partitioned into three temporal intervals according to the stimulus onsets (i.e., pre-stimulus, early post-stimulus, and late post-stimulus periods). For each window of interest, a novel metric—namely, the Multivariate Multiscale Multi-Frequency Entropy (M3FrEn)—was computed across 10 temporal scales. The obtained results showed significant main effects of *group* and *stimulus*, as well as a significant group-by-stimulus interaction across all scales, as assessed by linear mixed-effects models. Post hoc analysis revealed a significantly reduced stimulus-related entropy modulation in MCI subjects compared to healthy controls across several early post-stimulus scales, with the strongest effect at scale 6 (adjusted p=0.0189, Hedges’g=−2.88). An exploratory, fully nested subject-level classification analysis, in which feature selection was performed independently within each cross-validation fold, achieved an accuracy of approximately 92% with all classifiers, consistently relying on the early post-stimulus feature. These preliminary findings suggest that M3FrEn captures olfactory-related EEG alterations in MCI, providing proof-of-concept evidence for its potential as a candidate biomarker.

## 1. Introduction

Alzheimer’s disease (AD) and related neurodegenerative dementia represent a growing global health challenge due to population aging and the associated burden of disability and care needs [1]. AD is defined by the deposition of plaques of amyloid-beta (β-amyloid) and neurofibrillary tangles of τ protein, resulting in the impairment of synapses and subsequent death of neurons [2]. In this context, mild cognitive impairment (MCI) could be regarded as the stage of dementia in which there is noticeable cognitive impairment with normal functional abilities [3]. Current diagnostic workflows for AD and MCI rely mainly on clinical criteria and neuropsychological tests; however, standard screening tools such as the Mini-Mental State Examination (MMSE) and the Montreal Cognitive Assessment are limited by educational and cultural bias and often lack sensitivity to early or atypical presentations. More advanced biomarkers, including positron emission tomography and cerebrospinal fluid assays, can identify AD’s pathology, but they are invasive, costly, and unsuitable for large-scale screening [4,5]. Consequently, considering the irreversible nature of neurodegeneration, there is a need for accessible and sensitive biomarkers that enable early detection, particularly at the MCI stage, to support timely intervention and clinical trial stratification [6,7].

In this scenario, the olfactory system has attracted increasing attention as a biomarker of changes that occur in the early stages of AD [8]. Indeed, neuropathological and neuroimaging evidence indicates that the olfactory bulb, piriform and entorhinal cortices, and limbic structures are among the first regions to accumulate A β plaques and τ tangles and to undergo atrophy, in parallel with early synaptic dysfunction [9]. The clinical implication of such susceptibility is the onset of olfactory impairment, which often precedes memory loss and dementia. Specifically, odor identification impairment is highly prevalent in both MCI and AD [9,10], and longitudinal studies suggest that olfactory dysfunction may predict cognitive decline and conversion to AD [8]. These findings support olfactory impairment as a low-cost and potentially scalable prodromal marker [11].

Besides psychophysical tests, electroencephalography (EEG) has emerged as a non-invasive and accessible tool for investigating brain activity and supporting the development of digital biomarkers in neurodegenerative diseases [5,12]. Quantitative EEG (qEEG) studies, conducted in both resting-state and task-based paradigms, consistently show alterations in AD and MCI, including background slowing (i.e., increased δ and θ power, reduced α and β power), impaired and delayed event-related potentials, and widespread disruptions in functional connectivity [6,7,13,14]. These changes are interpreted as signatures of large-scale network disconnection and synaptic dysfunction, and they correlate with disease severity and risk of transition from MCI to AD [7]. Nonlinear measures of signal complexity and entropy further suggest a progressive reduction in EEG complexity from healthy aging to MCI and AD, consistent with loss of dynamical richness in cortical activity [15,16,17].

Within this framework, EEG can be specifically applied for investigating brain activity in response to olfactory stimulation [5,18,19]. Olfactory event-related potentials (OERPs) represent the most commonly adopted approach, typically characterized by an early sensory component (N1) followed by later cognitive components (e.g., P2/P3). Alterations in these components, including reduced amplitudes and prolonged latencies, have been reported in both AD and MCI [20]. Nevertheless, the existing literature has predominantly focused on resting-state EEG or broadband recordings, while olfactory-evoked responses remain comparatively underexplored in spite of their informative content. This is especially important in MCI, since minor modifications in the temporal characteristics of EEG activity can be regarded as an indicator of functional changes even when ERPs remain unchanged with respect to amplitude and latency measures, thus motivating the use of more sensitive descriptors of signal dynamics.

On the basis of these considerations, the present study proposes a systematic and rigorous framework for the analysis of olfactory-evoked EEG in MCI. The analysis was performed on a publicly available olfactory oddball EEG dataset [21], widely employed in the literature, with data cleaning and preprocessing procedures not always described in a standardized and comprehensive manner. For this reason, in this work, particular emphasis is placed on the implementation of a rigorous data quality control pipeline. Specifically, procedures for identifying noisy or corrupted channels and artifact-contaminated epochs (e.g., due to eye blinks) were synergistically applied, ensuring a reliable and reproducible analysis. This step was combined with a structured preprocessing strategy aimed at extracting functionally meaningful temporal segments, enabling a consistent characterization of stimulus-related brain dynamics. Within this controlled framework, Multivariate Multiscale Multi-Frequency Entropy (M3FrEn) [22] was investigated as a candidate neurophysiological marker for capturing olfactory-evoked EEG changes associated with MCI and supporting the discrimination between healthy subjects (HSs) and individuals with MCI. This recently introduced entropy-based measure is specifically designed to capture the multiscale, multichannel, and multi-frequency complexity of EEG signals. Unlike conventional approaches, typically limited to univariate, single-scale, or band-specific analyses, M3FrEn provides a more comprehensive representation of brain dynamics; thus, it could be suited to detecting subtle alterations associated with early neurodegenerative processes.

The objective of this study is twofold: First, the discriminative capability of M3FrEn features extracted from olfactory-evoked EEG signals is statistically evaluated in distinguishing healthy elderly subjects from MCI patients. Second, the effectiveness of these features is assessed within a machine learning framework, with a focus on subject-level classification. By integrating rigorous data curation, structured preprocessing, and advanced complexity analysis, the proposed framework aims to address a methodological gap in the literature and to provide a more reliable assessment of EEG-based biomarkers for the early detection of cognitive decline.

The remainder of this manuscript is organized as follows: Section 2 reviews related work on EEG biomarkers in AD and MCI, with an emphasis on olfactory paradigms and entropy-based metrics. Section 3 describes the dataset and the experimental methodology. Section 4 presents the statistical and classification results, while Section 5 discusses the findings in the broader context of neurodegenerative research and potential clinical applications. Finally, concluding remarks and directions for future work are provided.

## 2. Related Works

Quantitative EEG (qEEG) has been widely investigated as a non-invasive tool for detecting early neurophysiological alterations in AD and MCI [6]. Resting-state studies consistently report a slowing of background activity, with increased δ and θ power and reduced α and β power [13]. As for task-evoked qEEG, the oddball paradigm, in particular, is known to reveal smaller amplitudes and slower latencies in P3 components, as a result of inefficient attentional resource allocation and context updating in MCI and AD [7]. Connectivity analyses have also revealed reduced long-range coupling, disrupted small-world topology, and altered hubness, which reflect large-scale functional disconnectivity and synaptic impairments [7,13].

Nonlinear EEG measures of signal complexity and entropy also show a progressive decrease in the course from healthy aging to MCI and then to AD [15,16]. These features have been applied in various multi-class classifiers and were found to provide information complementary to spectral parameters [15,16,22]. Nevertheless, current applications predominantly use broad frequency ranges in resting-state paradigms, leaving task-evoked responses comparatively unexplored [16,23].

In particular, few works address the assessment of EEG complexity and entropy in relation to olfactory evoked potentials in MCI and AD, despite the recognized role of olfactory dysfunction as biomarker of the early stages of AD [11]. Specifically, in AD patients, odor-evoked olfactory event-related potentials (OERPs) revealed delayed latencies of P2 and P3 peaks compared to healthy controls; those metrics were correlated with AD severity [20]. Moreover, cross-modal paradigms of odor recognition demonstrated differences between ApoE ε4 carriers and non-carriers through their olfactory ERPs, suggesting sensitivity of olfactory ERPs to early AD [11]. At the prodromal stage, incidental olfaction in MCI during a face-processing task in which some faces were paired with an odor has revealed that healthy older adults show odor-related ERP differences in early (approximately 160–320 ms) and late (approximately 640–720 ms) windows, whereas MCI patients do not [24]. This pattern was interpreted as evidence that both subliminal and conscious olfactory processing are already impaired at the MCI stage.

Beyond time-domain components, oscillatory and connectivity changes during olfactory stimulation have been described. Analyses of olfactory-related frontal oscillations during an oddball task, using slow-gamma phase-locking values and θ/γ phase-amplitude coupling, have revealed distinct group differences between odor and rest conditions [25,26]. To exploit complexity differences in EEG signals associated with neurodegenerative disorders, in [21], a machine learning (ML) approach that relies on connectivity metrics, such as imaginary coherence, was used to classify patients with AD based on olfactory stimulation-induced EEG signals against healthy individuals. Exploiting the same dataset, a Transformer-based architecture integrating common spatial patterns, covariance-matrix tangent-space representations, and tunable Q-factor wavelet coefficients was proposed in [27], achieving classification accuracies above 90% in a three-class problem (HS, MCI, AD) [27,28,29]. In [30], entropy-based features—specifically, sample entropy at the Fz/Pz electrodes—are extracted from OERPs and used in ML classifiers, exhibiting an accuracy of 92.14 %.

In [31], continuous wavelet transform paired with the canonical correlation analysis constituted the main feature classification exploited for the classification task between HS and MCI, with an accuracy of 96.17 %.

Overall, the findings suggest that olfaction-induced EEG represents a good candidate for biomarker development in AD.

## 3. Materials and Methods

The proposed framework aims at assessing the value of M3FrEn as a neurophysiological marker able to discriminate between patients with MCI and HS. M3FrEn was chosen because it overcomes the main limitations of conventional EEG markers in the context of early neurodegenerative changes. Unlike univariate, single-band, or single-scale entropy measures, which capture only partial aspects of brain dynamics, M3FrEn jointly captures spatial, temporal, and spectral dimensions of EEG complexity. It exploits the multichannel nature of EEG to assess dependencies across electrodes, incorporates multiple temporal scales to capture short- and long-range correlations, and integrates multiple frequency bands to characterize band-specific dynamics. This comprehensive representation of brain dynamics makes M3FrEn particularly suited to detecting the subtle, heterogeneous alterations associated with early neurodegenerative processes.

**Figure 1:**
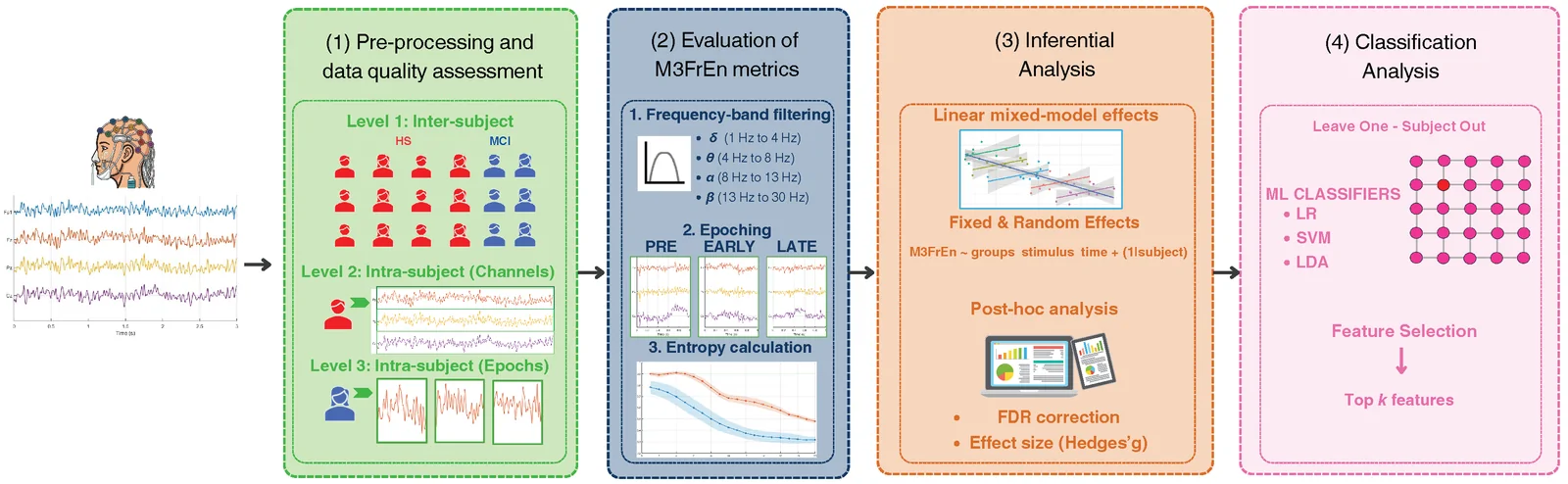
Schematic representation of the processing pipeline, including quality control, M3FrEn computation, statistical analysis, and exploratory classification.

The methodological workflow, represented in Figure 1, comprises four main steps: (i) hierarchical preprocessing and data quality assessment, (ii) computation of the M3FrEn entropy measure, (iii) inferential analysis based on linear mixed-effects modeling and post hoc pairwise contrasts, and (iv) exploratory subject-level classification analysis of EEG signals acquired during olfactory stimulus presentation.

### 3.1. Step #1: Preprocessing and Data Quality Assessment

#### 3.1.1. Dataset

The analysis was conducted on the publicly available EEG dataset comprising recordings from older adults classified into 7 subjects with MCI, 15 healthy subjects (HS), and 13 patients with AD [21]. MMSE scores, also provided with the dataset, are reported in Table 1 as a descriptive clinical characteristic of the included participants. Participants performed an olfactory oddball paradigm with frequent lemon stimuli (75% of trials) and rare rose stimuli (25% of trials). For each subject, 120 trials were recorded. Each trial lasted 10 s, including 2 s of olfactory stimulation followed by 8 s of rest. Signals were originally acquired at 2 kHz and downsampled to 200 Hz. As part of a conventional 19-channel 10–20 placement system, the EEG dataset provides recordings of the midline sites Fz, Cz, and Pz, along with the left frontopolar electrode Fp1. The data was filtered from 0.5 Hz to 40 Hz using a bandpass filter. Ocular artifacts were reduced by using Independent Component Analysis (ICA), removing a single component identified as corresponding to eye blinks, followed by manual visual inspection and rejection of residual noisy trials [21]. Additionally, the recorded trials are already pre-segmented into 3 s epochs, comprising 1 s of the pre-stimulus baseline and 2 s of post-stimulation.

For the present study, Fp1 was excluded due to potential contamination caused by eye movements. Moreover, only the HS and MCI subject groups were considered.

#### 3.1.2. Exclusion Criteria and Preprocessing

Initial exploratory inspection of the raw recordings revealed substantial artifact contamination in a considerable portion of the dataset, motivating the design of a hierarchical quality-control pipeline to identify and exclude subjects, channels, and epochs affected by poor signal quality or artifact contamination, based on robust statistical criteria [32,33,34]. The pipeline comprised a preliminary bitwise duplicate-check step (Level 0), conceptually distinct from the subsequent three-level hierarchical quality-control procedure (Levels 1–3) assessing signal quality at the subject, channel, and epoch levels, respectively. This procedure was designed to minimize data loss while preserving physiologically meaningful signals and was applied on top of the already ICA-cleaned data, constituting an additional quality-control layer.

#### Level 0: Duplicate-Record Check

As a preliminary data-integrity check, a bitwise comparison was performed across all recordings to identify potential duplicate files erroneously included in the public dataset.

#### Level 1: Inter-Subject Evaluation

The first level aimed to identify subjects whose recordings deviated markedly from the group distribution. For each subject, the variance of each channel was computed and then averaged across the three channels, yielding a subject-level variance index. In addition, the median absolute temporal gradient was calculated based on the median value of the absolute first difference, allowing us to capture any variability in the time signal and, hence, to detect any sections that were either flat, drifting slowly, or highly unstable [35]. Both indices were standardized across subjects using a robust *z*-score, based on the median and median absolute deviation (MAD) scaled by 1.5 to approximate the standard deviation in a normally distributed population [33]. Subjects exceeding a threshold of 3.0 on at least one index were excluded.

#### Level 2: Intra-Subject Channel Evaluation

The second level evaluated the quality of individual channels within each subject. Two criteria were considered—namely, flatline behavior and excessive noise—in agreement with recommendations from automated EEG cleaning frameworks [32,34].

Flatline channels were detected via a simple deviation criterion, which was the standard deviation of the respective channel. When the standard deviation of the signal dropped under a constant threshold of 0.5 μV, the channel was considered irrelevant. Abnormally noisy channels were detected by comparing a channel-wise metric with the median value of the same metric in the remaining two channels for each participant. The selected channel-wise metric was calculated using the power spectrum density (PSD) of the signals. For each channel, PSD estimates were obtained using the Fourier transform method on an epoch-by-epoch basis using the Hann window. A line noise ratio was computed as the mean power around 50 Hz, within ±1 Hz, divided by the mean PSD in the range 1 Hz to 40 Hz. This EEG band quantified the relative contribution of mains interference compared with the physiological EEG frequency range. The line noise ratio was then compared with the median ratio of the other available EEG channels from the same subject. Channels whose metric strongly deviated from this median ratio (over 5×median_ratio) were marked as noisy and excluded. To preserve consistent spatial information across subjects, any subject for whom one of the three channels of interest (Fz, Cz, or Pz) was excluded at this level was removed from the subsequent analysis.

#### Level 3: Epoch-Level Quality Assessment

The final level operated at the epoch scale to identify transient artifacts. Within each subject, epochs were defined using statistical measures like variance, kurtosis, and median gradient. Each descriptor was standardized using z-scores based on the subject-specific median and MAD, thereby accounting for inter-individual variability in EEG amplitude. Epochs whose descriptors exceeded an empirically predefined threshold ($z_{thresh}=3.0$) were rejected. Noisy epochs annotated in the original dataset were combined with those identified by the automated procedure. After this stage, the number of retained epochs varied across participants, with the minimum number of retained rose epochs across the final cohort equal to 9. To ensure a comparable contribution from every subject, a fixed number of epochs per condition was subsequently retained for all participants, thus preserving the original 3:1 lemon-to-rose stimulus ratio. More specifically, the first 30 lemon and first 9 rose epochs that survived the quality-control pipeline were retained for each subject.

Following this quality assessment, 9 subjects were excluded from the original HS group (1 at the preliminary Level 0 duplicate check, 7 at Level 1, and 1 at Level 2), while no MCI participants were excluded, yielding a final cohort of 6 HS and 7 MCI participants. Table 1 reports demographic and clinical characteristics of the included subjects, while Table A1 (in the Appendix A) reports the same characteristics for all 15 original HS participants, distinguishing included from excluded subjects.

**Figure 2:**
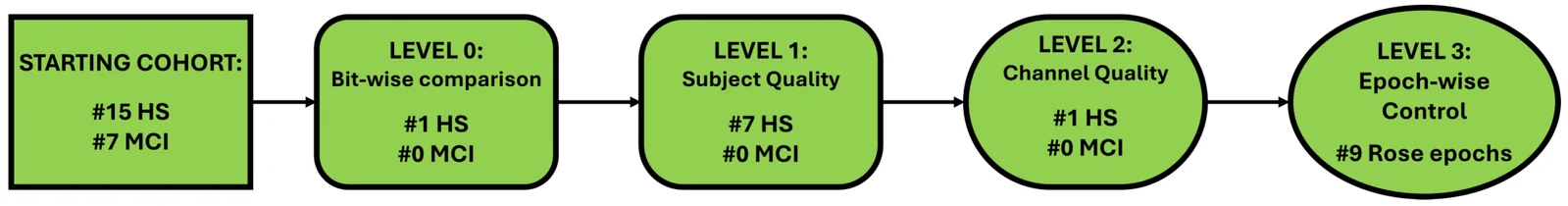
Quality-control workflow with the number of participants excluded at each level.

For each subject, 9 rose epochs and 30 lemon epochs were considered. Figure 2 summarizes the complete quality-control workflow, including the number of participants excluded at each level.

### 3.2. Step #2: Evaluation of the M3FrEn Metrics

Multivariate Multiscale Multi-Frequency Entropy (M3FrEn) was calculated for each subject. M3FrEn is an entropy-based measure that jointly captures spatial, temporal, and spectral dimensions of EEG complexity [22]. This method extends the multivariate multiscale entropy approach to characterize both intra- and inter-regional EEG complexity associated with pathological brain dynamics.

Spatial complexity is captured by exploiting the multichannel nature of EEG, assessing dependencies across the three midline electrodes (Fz, Cz, Pz). These three channels correspond to the standard configuration for recording olfactory event-related potentials [18]. Temporal dynamics is incorporated by coarse-graining the time series into non-overlapping windows across multiple scale factors in order to capture both short- and long-range correlations. Spectral information is also included by breaking the signals down into the EEG bands δ ([1–4] Hz), θ ([4–8] Hz), α ([8–13] Hz), and β ([13–30] Hz), and then calculating the entropy across these bands. The lower bound of the δ band was set to 1 Hz, rather than the conventional 0.5 Hz, to ensure a more stable filtering within the 3 s analysis window (approximately 3 cycles at 1 Hz).

**Figure 3:**
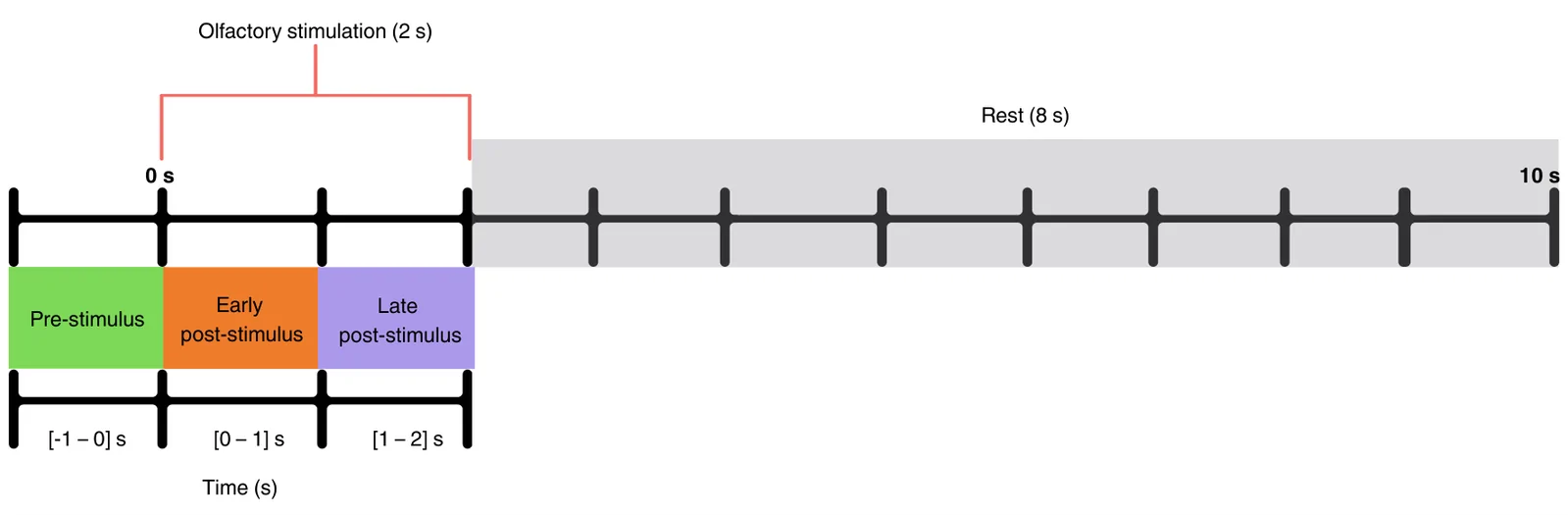
Schematic representation of the olfactory oddball EEG paradigm. Each trial lasted 10 s, with 2 s of olfactory stimulation (lemon: 75% of trials, rose: 25% of trials) followed by 8 s of rest. Time is expressed relative to olfactory stimulus onset (0 s). The 3 s analysis epoch spans from −1 s (start of the *pre-stimulus* baseline) to 2 s (end of the *late post-stimulus* window).

Specifically, for the present study, the pre-segmented 3 s epochs were bandpass-filtered into the four frequency bands. After the filtering stage, each epoch was partitioned into three time windows: *pre*-stimulus window (−1 s to 0 s), *early* post-stimulus window (0 s to 1 s), and *late* post-stimulus window (1 s to 2 s). The temporal structure of the paradigm and the three analysis windows are illustrated in Figure 3.

Mathematically, let *U* be the matrix variable representing the multivariate matrix, and each element $u_{h,c}$ of *U* is the bandpass-filtered signal at the frequency band h∈δ,θ,α,β and at the channel c∈Fz,Cz,Pz. For each time scale *S*, a coarse-grained representation $M_{h,c}^{S}$ is first obtained. Subsequently, for each band and channel, delay embedding vectors of dimension *m* are formed and concatenated across channels to give multivariate embedded vectors of length m·Z, where *Z* is the number of channels. Chebyshev distance is computed among these multivariate vectors, assessing their similarity. It averages out both temporal dynamics and spatial patterns into one scalar value for each frequency band. Therefore, a vector of inter-band distances is computed for each time pair: $d=\left[D_{\delta },D_{\theta },D_{\alpha },D_{\beta }\right]$, where each element corresponds to the largest divergence between embedded multichannel trajectories in a specific frequency band. Permutation patterns are constructed by ranking the elements of d in ascending order. Since g=4 bands, this will lead to a total of g!=24 different permutation patterns. Time and space are also incorporated into the embedding and multivariate distance calculation. The Shannon entropy $H_{p}$ of the resulting distribution is then normalized by the maximum possible entropy, ln(g!), where *g* is the number of considered bands, yielding

(1) $$M3FrEn\left(U,m,S,c\right)=\frac{H_{p}}{ln(g!)}$$

For this study, M3FrEn was computed for each EEG segment by using an embedding dimension m=2 and a time lag of τ=1, following the parameter settings validated in the original M3FrEn study [22]. Given the 200-sample length of each 1 s analysis window, the M3FrEn analysis was calculated considering 10 temporal scales, ensuring a minimum coarse-grained sequence length of 20 samples at every scale.

For each subject, stimulus condition, and time window, entropy values were averaged over all of the available epochs to obtain one value per condition. This aggregation over the subject turns the subject into the inferential unit and mitigates the danger of pseudo-replication.

### 3.3. Step #3: Inferential Analysis

#### 3.3.1. Linear Mixed-Effects Model

To evaluate differences in M3FrEn across experimental conditions, a linear mixed-effects model (LMM) was employed. LMMs are a regression-based models that combine fixed effects, representing population-level experimental factors, with random effects, accounting for subject-wise variability [36]. To account for multiple comparisons, Benjamini–Hochberg false discovery rate (FDR) correction was applied separately for each fixed effect (group, stimulus, time, and their two- and three-way interactions), each correction comprising the corresponding set of 10 *p*-values obtained across the 10 temporal scales (i.e., 7 separate FDR families, each of size 10). Effects surviving this analysis were further assessed via post hoc contrasts.

This method was selected to account for the nested structure of the data and the dependence among repeated measurements within subjects. The fixed effects were group (HS and MCI), stimulus (lemon and rose), and temporal window (pre, early, and late), as well as all pairwise and three-way interactions. Subject-specific variability was modeled as a random intercept term. Models were fitted via restricted maximum likelihood (REML) in MATLAB (R2024b, fitlme), using the software’s default covariance structure, comprising two variance components: the standard deviation of the random intercept (between-subject variability) and the standard deviation of the residuals (within-subject error). Random slopes were not included in addition to the random intercept, given the limited number of subjects per group (6 HS, 7 MCI), which would not have supported stable estimation of additional random-effects variance components. The resulting formulation was

(2) M3FrEn∼group×stimulus×time+(1|subjects)

The inclusion of time window as a fixed effect, together with its interactions with group and stimulus, allows the model to account for potential baseline pre-stimulus differences between groups.

Following the epoch-level quality-control procedure described in Section 3.1, each subject provided multiple epochs for each condition (30 lemon and 9 rose epochs). To mitigate for pseudo-replication and highlight inter-subject variability in the statistical analysis, epochs from the same condition were averaged across each temporal window before statistical testing. Hence, the individual was the main inferential unit of the analysis.

Normality of model residuals was assessed qualitatively via Q-Q plots for each fitted model. Homoskedasticity was assessed both qualitatively, via raw conditional residual-versus-fitted-value plots for each of the 10 scale-specific models, and quantitatively, via an exploratory Spearman correlation between residuals and fitted values. Across all scales, the residual-versus-fitted-value plots did not show a clear, systematic funnel-shaped or otherwise structured pattern, supporting the homoskedasticity assumption, although some heterogeneity in residual dispersion was visible at certain scales (e.g., scales 9–10), consistent with the comparatively smaller effective sample sizes at coarser scales.

Statistical significance of fixed effects and interactions was assessed by conducting analysis of variance (ANOVA). All analyses were performed in MATLAB (MathWorks, version 24.2).

#### 3.3.2. Post Hoc Analysis

Post hoc analyses were performed by using estimated marginal means to characterize significant main effects and interactions. Pairwise contrasts of condition (*group* × *stimulus* × *time*) were calculated based on the estimated marginal means. Within-group analyses compared lemon versus rose stimuli, while between-group analyses compared HS versus MCI for each stimulus and time window.

In addition, subject-level difference scores between rose and lemon conditions ($\Delta =M3{\mathrm{FrEn}}_{\mathrm{rose}}-M3{\mathrm{FrEn}}_{\mathrm{lemon}}$) were computed to quantify stimulus-specific modulation of complexity and were compared between groups using two-sample t-tests. For each of the 10 scales and each of the 3 temporal windows, this comparison was performed separately, yielding 30 statistical tests (10 scales × 3 windows) in total.

This analysis was performed on aggregated subject-level measures and was therefore independent of the LMM residual structure. To correct for multiple testing over scales and conditions, the FDR procedure was applied. Estimates of effect sizes were computed via Hedges’ *g*, which is the bias-corrected standardized mean difference.

### 3.4. Step #4: Classification Analysis

An exploratory subject-level classification was also performed on M3FrEn features to evaluate their discriminative power. Each subject was represented by a feature vector built from entropy differences between rare and frequent stimuli, considering the full set of 30 scale-by-window combinations (10 temporal scales × 3 time windows) as candidate features.

A nested leave-one-subject-out (LOSO) cross-validation scheme was adopted. In the outer loop, one subject was held out as the test case, and the remaining subjects formed the training set. Within each outer training set, an inner LOSO loop was used to rank the candidate scale-by-window features and to select the number of *k* retained features in the range 1–5 using univariate ranking on the training data only, ensuring that no information from the held-out test subject informed any stage of feature selection. Following feature selection, the training and test samples were normalized by *z*-score normalization, with the training set being used to estimate the mean and standard deviation.

Three linear classifiers were evaluated: logistic regression (LR), linear support vector machine (SVM), and linear discriminant analysis (LDA). These models were chosen for their robustness and because they are considered well suited to small-sample, high-dimensional scenarios. Predictions at the subject level for the outer folds were combined to compute confusion matrices and performance metrics. Classification performance was quantified in terms of accuracy (Acc), sensitivity, specificity, and balanced accuracy (BAcc), defined as

(3) $$\mathrm{Acc}=\frac{TP+TN}{TP+TN+FP+FN},$$

(4) $$\mathrm{Sensitivity}=\frac{TP}{TP+FN},$$

(5) $$\mathrm{Specificity}=\frac{TN}{TN+FP},$$

(6) $$\mathrm{BAcc}=\frac{\mathrm{Sensitivity}+\mathrm{Specificity}}{2},$$

where TP, TN, FP, and FN denote the numbers of true positives, true negatives, false positives, and false negatives, respectively.

To obtain a descriptive estimate of the variability in the performance metrics, a non-parametric bootstrap with 5000 resamples was applied to the subject-level predictions. The resulting distributions were summarized in terms of mean, standard deviation, and confidence intervals.

## 4. Results

**Figure 4:**
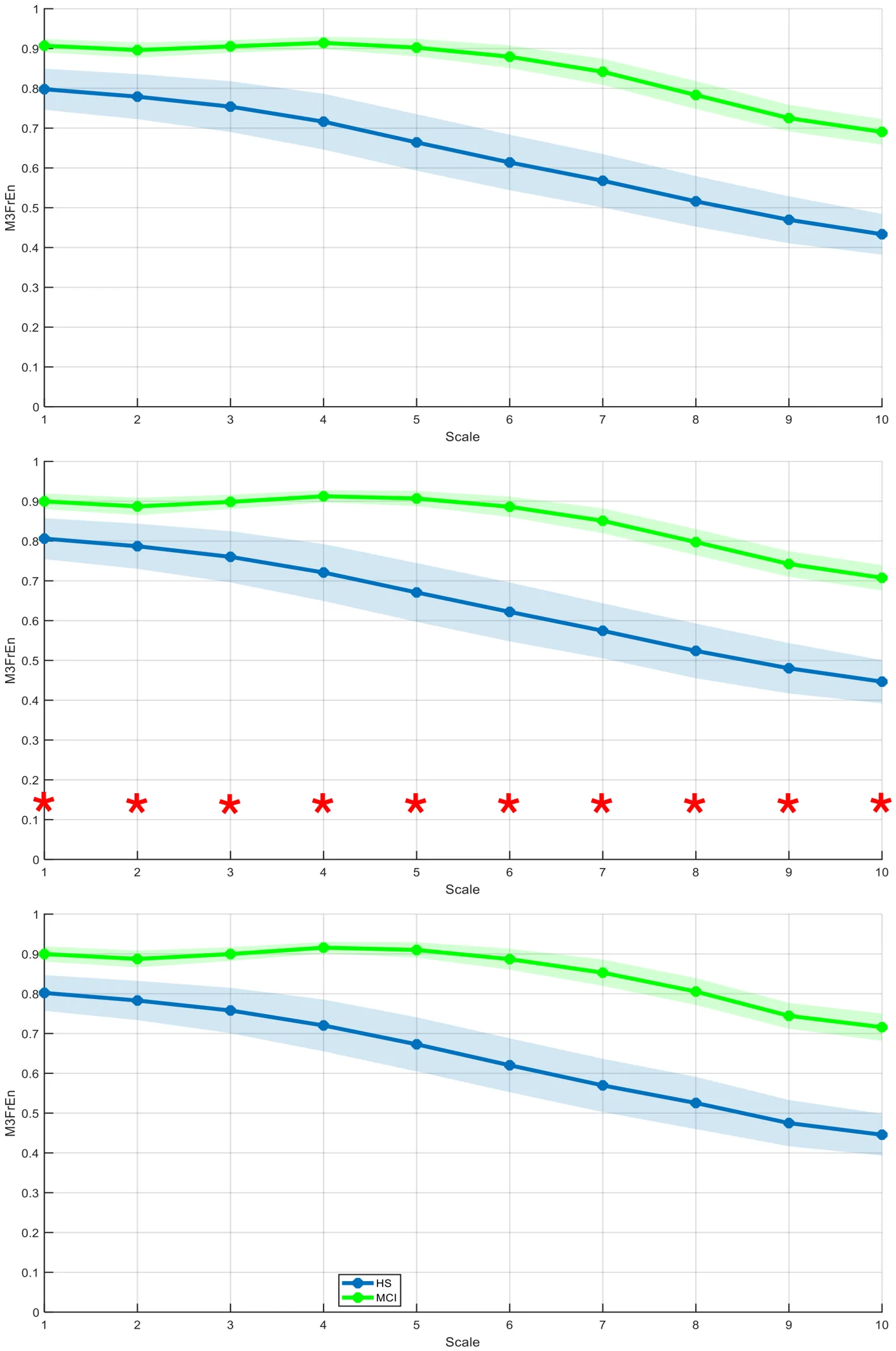
Mean M3FrEn profiles across temporal scales for healthy subjects (HS) and mild cognitive impairment (MCI) participants. Shaded areas represent standard deviations of the mean. Asterisks indicate scales at which the group difference is statistically significant. From the top to the bottom, pre-, early-, and late-stimulus time windows are represented, respectively.

The mean M3FrEn profiles at different temporal scales for HS and MCI, along with the standard deviation bands, are presented in Figure 4. MCI had a higher entropy than HS for almost all temporal scales in all panels, which implies that olfactory-evoked EEG is a less regular dynamical system in MCI. In both cases, M3FrEn tends to decrease as the scale increases, suggesting that both groups exhibit decreasing complexity at larger temporal scales. The variation around the average was still scarce, meaning that the group-level tendency was robust across scales.

### 4.1. Inferential Analysis

The linear mixed model’s results revealed significant main effects of group and stimulus after FDR correction. The *stimulus* effect was strongest on the low and middle scales, at about scales 1–6, where the smallest *p*-values were found. Conversely, there were no significant main effects of *time* on any scale. Specifically, for each subject, the features were computed as the difference in M3FrEn between the rare and common stimuli ($\Delta =M3{\mathrm{FrEn}}_{\mathrm{rose}}-M3{\mathrm{FrEn}}_{\mathrm{lemon}}$) at scales and time windows that revealed significant effects in the LMM. This design aimed to capture subject-specific, stimulus-related modulation of complexity in a compact form. For the three two-way interactions (*group* × *stimulus*, *group* × *time*, *stimulus* × *time*) and the three-way interaction (*group* × *stimulus* × *time*), the only term to survive FDR correction in each of the 10 scales was the *group* × *stimulus*. This finding suggests the modulation of stimulus-related M3FrEn between HS and MCI.

In order to better describe the significant *group* × *stimulus* interaction, post hoc simple main-effect analyses were conducted. For every participant and level of the scale, stimulation-related modulation was measured by the difference

$$\Delta_{\mathrm{rose}\text{-}\mathrm{lemon}}=M3{\mathrm{FrEn}}_{\mathrm{rose}}-M3{\mathrm{FrEn}}_{\mathrm{lemon}}.$$

Following FDR adjustment for multiple comparisons across scales, a significant group difference in $\Delta_{\mathrm{rose}\text{-}\mathrm{lemon}}$ was found at mainly scale 6 (FDR-adjusted p=0.0189). At this scale, HS demonstrated a negative rose-minus-lemon modulation [negative $\Delta_{\mathrm{rose}\text{-}\mathrm{lemon}}$] equal to −0.062±0.030, and MCI showed a minimal positive modulation [positive $\Delta_{\mathrm{rose}\text{-}\mathrm{lemon}}$] equal to 0.015±0.019, representing a great effect size (Hedges’ g=−2.88). Table A2 reports approximated ${\eta_{p}}^{2}$ for the group × stimulus effect. Figure 4 illustrates the significative temporal scales on the basis of $\Delta_{\mathrm{rose}\text{-}\mathrm{lemon}}$ across the three temporal windows, showing the separation between groups in the early post-stimulus interval. By contrast, no significant effects were observed for either the pre-stimulus or the late post-stimulus window after FDR correction. The most significant contrast was observed at scale 6 (early window), followed by scales 3 and 4. Scales 1, 2, 5, 7, 8, 9, and 10, also within the early time window, likewise reached significance (corrected p<α). All reported significant contrasts survived FDR correction applied to the post hoc analysis.

### 4.2. Classification Analysis

**Figure 5:**
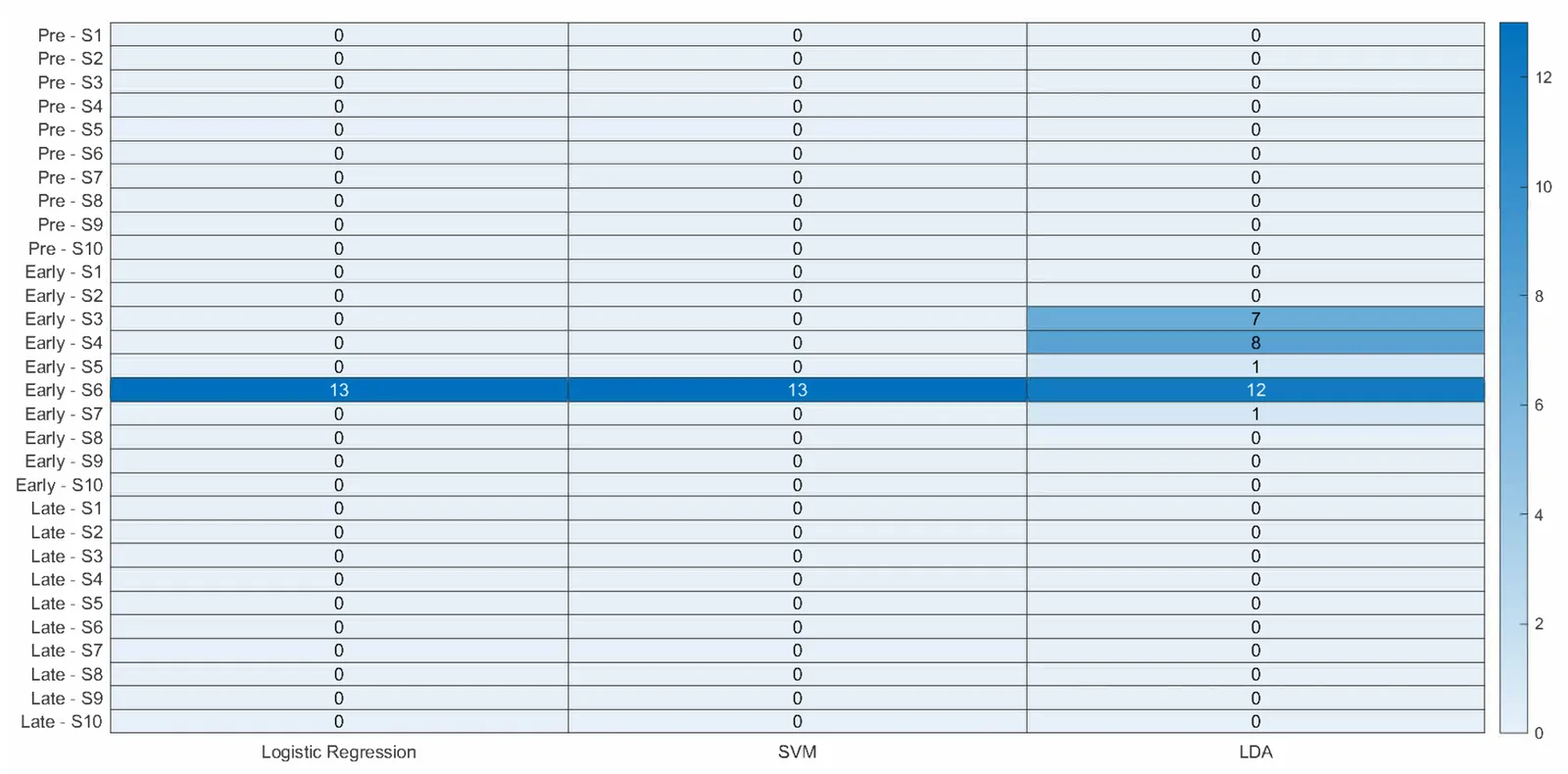
Feature-selection frequency across the 13 outer LOSO folds for each of the 30 candidate scale-by-window features (10 temporal scales × 3 time windows: pre-, early-, and late post-stimulus), for the LR, SVM, and LDA classifiers.

As an exploratory, proof-of-concept extension of the inferential analysis, subject-level classification was performed to assess whether the identified entropy-based contrasts carry discriminative information beyond group-level statistical significance. Figure 5 shows the number of outer LOSO folds (out of 13) in which each candidate feature was selected across the three classifiers. Further details regarding the LOSO CV are provided in Table A3. The scale-6, early-window feature was by far the most frequently selected across all models, being chosen in 13 out of 13 folds for both LR and SVM, and 12 out of 13 folds for LDA. All other selected features were also confined to the early post-stimulus window, while no pre-stimulus or late post-stimulus feature was ever selected. This indicates that the discriminative signal identified in the inferential analysis (Section 4.1) is consistent.

To evaluate the stability of the classification performance, a non-parametric bootstrap with 5000 resamples was applied to the subject-level predictions from each outer fold. All three models achieved comparable performance. For LR, the mean bootstrap accuracy was (92.3±7.4)% (95% CI: 76.9–100.0%) and the mean balanced accuracy was (91.6±8.2)% (95% CI: 75.0–100.0%), with sensitivity and specificity of (99.8±1.4)% and (83.2±16.3)%, respectively. Similar results were obtained for the SVM classifier, with a mean accuracy of (92.2±7.5)% and a mean balanced accuracy of (91.5±8.3)%. LDA achieved a comparable mean accuracy of (92.1±7.4)% with a mean balanced accuracy of (91.5±8.2)%, as reported in Table 2. These values represent bootstrap means and, therefore, may differ slightly from the performance metrics computed directly from the observed LOSO predictions. Based on the confusion matrices in Table 3, all three classifiers correctly classified 12 out of 13 subjects, corresponding to an observed accuracy of 92.3%.

Confusion matrices (Table 3) confirmed that all three classifiers correctly predicted 5 out of 6 HS and 7 out of 7 MCI participants.

For all models, the confidence intervals for the performance metrics remained wide (e.g., accuracy CI: 76.9–100.0% for LR), indicating that the results may be influenced by the small sample size, and further highlighting the exploratory nature of these findings.

## 5. Discussion

The mixed-effects analysis revealed significant effects of *group* and *stimulus* on M3FrEn, together with a robust *group* × *stimulus* interaction across temporal scales, indicating that olfactory stimulation modulates EEG complexity differently in HS and MCI. The lack of a significant *time* effect in the above analyses may indicate that the detected differences are not mainly due to the switching among the pre–early–late windows. Rather, they seem to indicate a more global stimulus-related change in complexity.

An early post-stimulus window maximal group difference was observed at scale 6, showing the strongest post hoc simple effect (smallest FDR-adjusted *p*-value and largest Hedges’ g). At this scale, HS displayed decreased entropy for the rare stimulus (rose) compared to the frequent one (lemon), while MCI exhibited a reverse trend.

The direction of this effect appears to contrast with the majority of previous EEG complexity studies in AD and MCI, which typically report reduced entropy or signal complexity in patients relative to HS [15,37]. Nevertheless, this apparent discrepancy may be explained by different points. First, most prior findings of reduced complexity derive from resting-state recordings, whereas the present study analyzed task-evoked EEG during an active olfactory oddball paradigm, which recruits additional attentional and executive resources not engaged at rest. Second, increased EEG complexity has been reported in early or preclinical stages of neurodegeneration and interpreted as evidence of compensatory neural recruitment, with compensation subsequently failing as the pathological burden increases [38,39,40]. Therefore, our finding may reflect a stage-dependent, non-monotonic trajectory of complexity across the HS–MCI–AD continuum, rather than a uniform reduction. Third, given the early involvement of olfactory-related cortical structures in AD’s pathology, a compensatory response may be particularly pronounced when probed through an olfactory paradigm compared to other sensory modalities. Finally, M3FrEn measures a construct distinct from the univariate, single-band, or single-scale entropy indices typically used in the AD and MCI literature [22]; as a result, an increase in M3FrEn does not necessarily entail an increase along every dimension of complexity captured by simpler measures.

While scale 6 (early window) showed the strongest group difference, several other early-window scales (3, 4, 1, 2, 5, 7, 8, 9, and 10) also survived FDR correction in the post hoc analysis, indicating that the group × stimulus effect is not confined to a single, isolated scale but spans a broad portion of the considered scale range. The physiological interpretation of this pattern nonetheless remains open. Following the coarse-graining definition of temporal scale adopted in [22], each temporal scale *S* corresponds to an effective sampling rate of $f_{s}/S$; scale 6, at which the strongest effect was observed, corresponds to an effective temporal resolution of approximately 30 ms, occupying a central-to-upper position within the validated scale range, while the broader set of significant scales spans a wider range of effective temporal resolutions. However, given the exploratory nature of this study, the small sample size, and the absence of concurrent measures (e.g., source localization or oscillatory phase analysis) able to directly link these temporal scales to specific olfactory neural generators, clarifying the physiological substrate of scale-dependent entropy modulations in olfactory-evoked EEG represents an important direction for future studies with larger cohorts. Nonetheless, this scale-specific pattern underlines the importance of a multiscale approach, as varying temporal scales may highlight distinct alterations along the neurodegenerative continuum from healthy aging to MCI and AD [15,16].

The results of the classification suggest that a small number of M3FrEn-derived features, consistently identified as the scale-6 early post-stimulus feature (Section 4), could contain subject-level discriminative information related to olfactory stimulation. All classifiers achieved accuracies and balanced accuracies of approximately 92%, with comparable performance across models, all correctly classifying 5 out of 6 HS and 7 out of 7 MCI participants. Based on the observed LOSO predictions, all three classifiers achieved a sensitivity of 100.0% (7/7 MCI subjects correctly classified) and a specificity of 83.3% (5/6 HSs correctly classified). The bootstrap estimates are very close to these values, with mean sensitivity of 99.8% for LR and 100.0% for SVM and LDA, and mean specificity ranging from 83.0% to 83.2% across models. The use of a fully nested LOSO cross-validation scheme, in which the entire feature-selection procedure—including the choice of scale and time window—was performed exclusively within the training folds, was aimed at minimizing information leakage and overfitting. Nonetheless, the small number of subjects limits the extent to which these results can support strong generalizability claims, and the present classification analysis should be regarded as an exploratory and proof-of-concept assessment.

Table 4 compares the present findings with recent work using the same publicly available olfactory EEG dataset. As also stated in Section 2, reported classification accuracies in the literature range from approximately 91% to 96%. The accuracy achieved in this study (92.3% observed LOSO accuracy; 92.2–92.3% bootstrap mean across classifiers) is therefore comparable to previously reported values. Several differences with respect to prior studies should nonetheless be noted. First, most prior studies rely on the preprocessing provided with the original dataset alone, without additional quality control, whereas the present work applies a considerably more conservative, hierarchical quality-control pipeline, substantially reducing the final cohort size and, consequently, statistical power. Second, prior approaches generally employ higher-dimensional or learned feature representations, whereas the present study deliberately relies on a single, interpretable entropy-based feature per subject, prioritizing physiological interpretability over predictive performance. Third, validation strategies are not consistently reported across prior studies, making it difficult to rule out subject-level information leakage as a contributing factor to their higher accuracies, whereas the nested leave-one-subject-out scheme adopted here is specifically designed to minimize this risk. Finally, several prior studies address the three-class HS/MCI/AD problem or the comparatively more separable HS/AD contrast, rather than the more subtle HS/MCI distinction targeted in this work.

Overall, the obtained results suggest that M3FrEn may be a candidate measure that can capture changes in olfactory-related EEG complexity at the MCI stage and, thus, offers a well-structured framework for further studies involving a larger number of subjects. However, it is important to point out three limitations:

First, the sample size available for statistical inference was limited: the hierarchical quality-control procedure adopted to mitigate substantial noise and artifacts in the EEG recordings caused a considerable reduction in sample size, particularly affecting the HS group, and constrained the overall statistical power of the analysis. After correcting for multiple comparisons, although all 10 early-window scale-by-window contrasts survived FDR correction, with the strongest effect at scale 6 ($\Delta_{\mathrm{rose}\text{-}\mathrm{lemon}}$ at scale 6 in the early window), these results should be interpreted with caution given the limited cohort size. A sensitivity analysis excluding the δ band from the M3FrEn computation resulted in the loss of the group × stimulus interaction, indicating that δ-band activity is necessary for the observed effect; this is consistent with the well-established role of δ-band slowing as a hallmark of large-scale network disruption in AD and MCI [6,7,13,14] (Table A4). Future studies with larger cohorts will be needed to replicate and extend these findings.

Second, the interpretation of the rose–lemon modulation is constrained by the structure of the original paradigm [21], in which odor identity is structurally confounded with stimulus role. Indeed, lemon was always the frequent/standard stimulus (75%) and rose always the rare/deviant one (25%)—a pairing never reversed across trials or participants. This confound extends beyond stimulus probability, as rose and lemon also differ intrinsically in perceptual dimensions such as intensity, trigeminal component, familiarity, and pleasantness, none of which were recorded in the original dataset, which only provides odor labels and EEG recordings. Thus, in light of that, the rose–lemon modulation should therefore be regarded as a stimulus-evoked complexity index rather than evidence of a pure deviant-processing effect. Nevertheless, its preliminary analysis as a promising biomarker does not depend on isolating the underlying driver, as the central finding is that HS and MCI participants differentially modulate olfactory-evoked EEG complexity between the two stimuli. Finally, the present study analyzed entropy solely using three midline electrodes (Fz, Cz, Pz), a constraint imposed by the available dataset [21]. While these sites are the classical locations of maximal amplitude for the evoked-potential components elicited by oddball paradigms [18], and M3FrEn’s multivariate formulation does not, in principle, require dense spatial sampling to extract multichannel complexity information, the actual impact of this restricted montage on the sensitivity of the proposed approach was not empirically evaluated. This constitutes a limitation of the present work, and denser electrode arrays should be considered in future studies to assess whether broader spatial sampling further improves the detection of olfactory-evoked entropy alterations.

Despite the constraints imposed by the available dataset and the small sample, the present findings suggest that M3FrEn is a potential measure that can capture changes in olfactory-related EEG complexity at the MCI stage and, thus, offers a well-structured framework for further studies involving a larger number of subjects.

## 6. Conclusions

This study provides preliminary proof-of-concept evidence that entropy-based analysis of olfactory-evoked EEG may capture group differences between cognitively healthy older adults (healthy subjects, HS) and individuals with MCI. The proposed pipeline integrates a hierarchical quality-control procedure with multiscale entropy estimation and inferential analysis. This methodological step was essential to support a more robust detection of stimulus-related EEG complexity modulations in the presence of substantial noise and low-density EEG recordings from the public dataset. The results indicate that M3FrEn is sensitive to olfactory-related changes in brain dynamics and may potentially aid in identifying early cognitive decline. The inferential analysis, which involved multiple temporal scales and experimental conditions, revealed a significant group-by-stimulus interaction, with all 10 scale-specific contrasts in the early post-stimulus window remaining significant after correction for multiple comparisons, and the strongest effect observed at scale 6. The exploratory classification analysis further suggests that a small subset of entropy-based features contain discriminatory information in the olfactory condition, achieving classification accuracy of 92.3%, 92.2%, and 92.1% for LR, SVM, and LDA, respectively. Overall, these preliminary findings provide proof-of-concept evidence for the potential of multiscale entropy as a candidate biomarker for MCI, and they motivate its validation in larger, independent cohorts.

In future work, to further corroborate the present findings, a dedicated experimental campaign is being planned to collect olfactory-evoked EEG data from larger cohorts of HS, MCI, and AD subjects, together with relevant clinical (e.g., MMSE or PET analysis) and demographic factors (e.g., sex, age, educational level). In doing that, the protocol will adopt a multichannel acquisition and a balanced design, alternating the standard/deviant role across odors and/or sessions, in combination with psychophysical ratings of intensity, familiarity, and pleasantness for each odor and participant. This will enable a factorial sensitivity analysis to independently estimate the relative contributions of stimulus probability and odor-specific perceptual dimensions. Finally, the present analysis relied on previously validated values for the embedding dimension, time delay, and scale range, without an independent sensitivity analysis over alternative parameter settings; assessing the robustness of the present findings to different values of *m* and τ represents a significant direction for future work.

## Figures and Tables

**Table 1:** Demographic and clinical characteristics of the 13 participants (6 HS, 7 MCI) included in the final analysis, based on metadata provided in [21].

Group	Subject ID	Age Range (Years)	Sex	MMSE Score
HS	#1	75–80	Male	27
HS	#2	70–75	Male	30
HS	#6	60–65	Female	26
HS	#8	55–60	Female	27
HS	#12	65–70	Female	21
HS	#13	60–65	Male	28
MCI	#1	55–60	Male	22
MCI	#2	70–75	Female	22
MCI	#3	60–65	Male	21
MCI	#4	70–75	Male	21
MCI	#5	70–75	Female	25
MCI	#6	60–65	Female	27
MCI	#7	70–75	Female	28

**Table 2:** Performance of the LR, SVM, and LDA classifiers under the fully nested feature-selection scheme. Values are reported as the mean ± standard deviation across 5000 bootstrap resamples of the subject-level LOSO predictions.

Model	Accuracy (%)	Balanced Accuracy (%)	Sensitivity (%)	Specificity (%)
LR	92.3 ± 7.4	91.6 ± 8.2	99.8 ± 1.4	83.2 ± 16.3
SVM	92.2 ± 7.5	91.5 ± 8.3	100.0 ± 0.0	83.0 ± 16.7
LDA	92.1 ± 7.4	91.5 ± 8.2	100.0 ± 0.0	83.0 ± 16.3

**Table 3:** Subject-level confusion matrices for the LR, SVM, and LDA classifiers based on the observed LOSO predictions. Rows indicate true classes and columns predicted classes.

Model	True Class	HS	MCI
LR	HS	5	1
	MCI	0	7
SVM	HS	5	1
	MCI	0	7
LDA	HS	5	1
	MCI	0	7

**Table 4:** Comparison of recent works using the same publicly available olfactory EEG dataset [21], including preprocessing, extracted features, validation strategy, and classification performance.

Study	Preprocessing	Extracted Features	Validation Strategy	Reported Performance
Cansiz et al. (2025) [27]	Preprocessing provided with the original public dataset	Common spatial pattern, covariance-matrix tangent-space, and tunable Q-factor wavelet transform features	Subject-wise	93.14% Accuracy (HS vs. MCI vs. AD)
Riaz et al. (2025) [31]	Preprocessing provided with the original public dataset	Continuous wavelet transform, spectral pattern grouping, and canonical correlation analysis features	n.a.	96.17% Accuracy (HS vs. MCI)
Joseph et al. (2026) [30]	Preprocessing provided with the original public dataset	Sample entropy (Fz and Pz channels)	n.a.	92.14% Accuracy (HS vs. AD)
Albaidhani et al. (2026) [29]	Preprocessing provided with the original public dataset	Deep features extracted from continuous wavelet transform and short-time Fourier transform images	Subject-wise	91.43% Accuracy (HS vs. MCI vs. AD)
Sahu et al. (2026) [28]	Preprocessing provided with the original public dataset; sliding-window segmentation	n.a.	Subject-wise	91.50% Accuracy (HS vs. MCI vs. AD)
Present study	Hierarchical, three-level quality-control pipeline (subject, channel, and epoch levels)	Multivariate multiscale multi-frequency entropy ($\Delta_{\mathrm{rose}\text{-}\mathrm{lemon}}$, scale 6, early post-stimulus window)	Nested leave-one- subject-out	92.3%Accuracy (HS vs. MCI)

## Data Availability

The data analyzed in this study are multichannel EEG recordings from a publicly available dataset provided by Sedghizadeh et al. [21]. The dataset can be accessed via https://data.mendeley.com/datasets/sgzbgwjfkr/5 (accessed on 6 September 2026).

## Appendix A

**Table A1:** Demographic characteristics of all 15 original healthy subjects (HS), distinguishing participants included in and excluded from the final analysis, based on the MMSE metadata provided with the original public dataset [21].

Subject ID	Age Range (Years)	Sex	MMSE Score	Status	Exclusion Level
#1	75–80	Male	27	Included	–
#2	70–75	Male	30	Included	–
#3	65–70	Female	26	Excluded	Level 1
#4	60–65	Female	30	Excluded	Level 1
#5	70–75	Male	21	Excluded	Level 1
#6	60–65	Female	26	Included	–
#7	65–70	Male	29	Excluded	Level 1
#8	55–60	Female	27	Included	–
#9	70–75	Female	23	Excluded	Level 1
#10	70–75	Male	26	Excluded	Level 1
#11	70–75	Male	26	Excluded	Level 1
#12	65–70	Female	21	Included	–
#13	60–65	Male	28	Included	–
#14	70–75	Female	23	Excluded	Level 0
#15	75–80	Female	26	Excluded	Level 2

**Table A2:** Approximated ${\eta_{p}}^{2}$ for the group × stimulus effect.

Scale	${\eta_{p}}^{2}$
1	0.095
2	0.093
3	0.100
4	0.111
5	0.102
6	0.108
7	0.098
8	0.080
9	0.134
10	0.069

**Table A3:** Classification report, for each classifier involved, resuming the fold number ID for the LOSO CV cycle, the subject held out, the number of selected features k for the classification specifying (which are selected), and then the predicted class for the specific fold.

**Support Vector Machine**					
**Fold**	**Held-Out Subject**	**True Class**	**Selected Feature(s) k**	**Scale-Window**	**Predicted Class**
1	HS#1	HS	1	6-Early	HS
2	HS#2	HS	1	6-Early	HS
3	HS#3	HS	1	6-Early	HS
4	HS#4	HS	1	6-Early	HS
5	HS#5	HS	1	6-Early	MCI
6	HS#6	HS	1	6-Early	HS
7	MCI#1	MCI	1	6-Early	MCI
8	MCI#2	MCI	1	6-Early	MCI
9	MCI#3	MCI	1	6-Early	MCI
10	MCI#4	MCI	1	6-Early	MCI
11	MCI#5	MCI	1	6-Early	MCI
12	MCI#6	MCI	1	6-Early	MCI
13	MCI#7	MCI	1	6-Early	MCI
**Logistic Regression**					
**Fold**	**Held-Out Subject**	**True Class**	**Selected Feature(s) k**	**Scale-Window**	**Predicted Class**
1	HS#1	HS	1	6-Early	HS
2	HS#2	HS	1	6-Early	HS
3	HS#3	HS	1	6-Early	HS
4	HS#4	HS	1	6-Early	HS
5	HS#5	HS	1	6-Early	MCI
6	HS#6	HS	1	6-Early	HS
7	MCI#1	MCI	1	6-Early	MCI
8	MCI#2	MCI	1	6-Early	MCI
9	MCI#3	MCI	1	6-Early	MCI
10	MCI#4	MCI	1	6-Early	MCI
11	MCI#5	MCI	1	6-Early	MCI
12	MCI#6	MCI	1	6-Early	MCI
13	MCI#7	MCI	1	6-Early	MCI
**Linear Discriminant Analysis**					
**Fold**	**Held-Out Subject**	**True Class**	**Selected Feature(s) k**	**Scale-Window**	**Predicted Class**
1	HS#1	HS	3	(6, 3, 4)-Early	HS
2	HS#2	HS	2	(4, 5)-Early	HS
3	HS#3	HS	1	6-Early	HS
4	HS#4	HS	3	(6, 3, 4)-Early	HS
5	HS#5	HS	1	6-Early	MCI
6	HS#6	HS	3	(6, 3, 4)-Early	HS
7	MCI#1	MCI	3	(6, 3, 4)-Early	MCI
8	MCI#2	MCI	3	(6, 3, 4)-Early	MCI
9	MCI#3	MCI	3	(6, 3, 4)-Early	MCI
10	MCI#4	MCI	3	(6, 3, 4)-Early	MCI
11	MCI#5	MCI	1	6-Early	MCI
12	MCI#6	MCI	2	(6, 7)-Early	MCI
13	MCI#7	MCI	1	6-Early	MCI

**Table A4:** Reported values for the post hoc analysis, examining all three cases: without considering the delta band in the filtering process, considering the high delta band (1–4) Hz, and the canonical delta band (0.5–4) Hz.

	Early Scales	Without Delta Band	With Delta Band (1–4) Hz	With Delta Band (0.5–4) Hz
FDR-adjusted *p*-value	1	0.578	0.0225	0.0282
	2	0.578	0.0234	0.0282
	3	0.578	0.0225	0.0280
	4	0.578	0.0225	0.0280
	5	0.578	0.0234	0.0332
	6	0.578	0.0189	0.0104
	7	0.578	0.0225	0.0282
	8	0.578	0.0225	0.0377
	9	0.578	0.0234	0.0472
	10	0.578	0.0234	0.0377
Hedges’ g value	1	−0.539	−1.87	−2.05
	2	−0.586	−1.95	−2.23
	3	−0.776	−2.18	−2.46
	4	−0.965	−2.27	−2.41
	5	−0.752	−2.03	−1.97
	6	−1.262	−2.88	−2.73
	7	−1.179	−1.95	−1.99
	8	−0.742	−1.75	−1.50
	9	−0.684	−1.61	−1.50
	10	−0.796	−1.78	−1.33
